# Evaluation of Resistance of Eleven Maize Races (*Zea mays* L.) to the Red Spider Mite (*Tetranychus merganser*, Boudreaux)

**DOI:** 10.3390/plants11111414

**Published:** 2022-05-26

**Authors:** Mario Rocandio-Rodríguez, Jorge Ariel Torres-Castillo, María Cruz Juárez-Aragón, Julio Cesar Chacón-Hernández, Yolanda del Rocio Moreno-Ramírez, Sandra Grisell Mora-Ravelo, Rafael Delgado-Martínez, Agustín Hernández-Juárez, Rapucel Tonantzin Quetzalli Heinz-Castro, Francisco Reyes-Zepeda

**Affiliations:** 1 Institute of Applied Ecology, Universidad Autónoma de Tamaulipas, Ciudad Victoria 87019, Mexico; mrocandio@docentes.uat.edu.mx (M.R.-R.); joatorres@docentes.uat.edu.mx (J.A.T.-C.); mcjuarez@uat.edu.mx (M.C.J.-A.); yrmoreno@docentes.uat.edu.mx (Y.d.R.M.-R.); sgmora@docentes.uat.edu.mx (S.G.M.-R.); freyes@docentes.uat.edu.mx (F.R.-Z.); 2 Faculty of Engineering and Sciences, Universidad Autónoma de Tamaulipas, Ciudad Victoria 87149, Mexico; rdelgado@docentes.uat.edu.mx; 3 Parasitology Department, Universidad Autónoma Agraria Antonio Narro, Saltillo 25315, Mexico; chinoahj14@hotmail.com; 4 Faculty of Agronomy and Veterinary, Universidad Autónoma de San Luis Potosí, Soledad de Graciano Sánchez 78321, Mexico; rapucel.heinz@uaslp.mx

**Keywords:** oviposition, feeding damage, population growth, resistance, immature mites, mortality, secondary metabolites, stomata, leaf thickness

## Abstract

At least 59 maize races (*Zea mays* L.) have been registered in Mexico. The feeding damage caused by insects and mites to maize crops generates up to ~30% of maize yield losses. Spider-mite-resistant plants are needed. The red spider mite, *Tetranychus merganser* Boudreaux (Acari: Tetranychidae), is distributed in the United States, China, Mexico, and Thailand. It is considered a potential pest in Mexican agriculture. The aim of this study was to evaluate the resistance mechanisms (antixenosis and antibiosis) of 11 native maize populations, representative of each race of maize grown in Tamaulipas, Mexico, to *T. merganser* under laboratory conditions. The aim was also to obtain information on the chemical composition and some morphological characteristics of these maize races and to identify resistant maize races for incorporation into a breeding program. Antixenosis was assessed by non-preference for oviposition and feeding. Antibiosis was measured by growth rate (ri). The presence of secondary metabolites in the 11 maize races were different. In the 11 maize races, quantitative analysis of total phenol concentration, total flavonoid concentration, and antioxidant capacity were significantly different. The multivariate analysis of variance showed that there is evidence of antixenosis noted by maize race differences in egg laying and percentage feeding damage but not of antibiosis noted by growth rate. Red spider mites laid significantly more eggs on the Celaya (24 h: 25.67 ± 17.04, 48 h: 42.67 ± 26.86, 72 h: 49.33 ± 28.54) race than on Raton (24 h: 7.00 ± 5.00, 48 h: 12.67 ± 8.02, 72 h: 14.67 ± 9.29) and Elotes Occidentales × Tuxpeño (24 h: 9.67 ± 5.85, 48 h: 15.33 ± 10.69, 72 h: 17.67 ± 10.97) races. However, the growth rate and mortality of *T. merganser* in the 11 corn races were similar. The Vandeño (24 h: 11.67 ± 2.89, 48 h: 27.67 ± 7.64, 72 h: 30.00 ± 18.03) and Tabloncillo × Tuxpeño (24 h: 18.33 ± 7.64, 48 h: 25.00 ± 8.66, 72 h: 25.00 ± 8.66) races were the most resistant to red spider mite damage, whereas the most susceptible race was Celaya (24 h: 26.67 ± 15.28, 48 h: 48.33 ± 29.30, 72 h: 65.00 ± 30.00). Further analysis by PCA at 24, 48, and 72 h found the Celaya race positively correlated to growth rate and oviposition of *T. merganser* and to a lesser extent with the percentage of feeding damage, suggesting that the Celaya race was most susceptible to *T. merganser*. At 24 h, the Vandeño race was most resistant, given a negative correlation to growth rate and oviposition by *T. merganser*. The PCA at 48 and 72 h noted the Elotes Occidentales × Tuxpeño race was most resistant to red spider mite, with negative relationships to growth rate and oviposition and, to a lesser extent, to feeding damage. This resistance is due to the differences in both its morphological characteristics and the secondary metabolites present in their leaves.

## 1. Introduction

In Mexico, there are more than 59 races of maize (*Zea mays* L.), each associated with specific origins, distribution, and geographic regions [1]. These maize races present genetic diversity related to their biological and anthropocentric processes, their selection, their adaptation, and the cultivation of different niches and ecological biotypes in various socioeconomic conditions, which has generated a wide diversity of maize. This is a fundamental feature of programs and strategies to improve agricultural challenges [2,3].

World corn production for the 2020/2021 agricultural cycle was 1120.65 million metric tons [4]. Corn production is affected between 6% and 19% due to herbivore by arthropod pests [5]. However, climate change has increased the metabolic rates and winter survival of arthropods, which coupled with a global average surface temperature increase of 2 °C may cause maize yield losses of up to 30% of damaged crops caused by insects and mite pests [6,7]. Corn cultivation is susceptible to attack by different insect pests, such as *Ostrinia nubilalis* Hübner (Lepidoptera: Crambidae), *Spodoptera frugiperda* Smith (Lepidoptera: Noctuidae), *Mythimna separata* Walker (Lepidoptera: Noctuidae), and *Rhopalosiphum maidis* Fictch (Hemiptera: Aphididae) [8,9,10]. Regarding the damage caused by feeding of spider mites (Acari: Tetranychidae) to corn crops, around 43 species have been reported, clustered into 5 genera, *Eutetranychus* (Banks) (2 spp.), *Oligonychus* (Berlese) (19 spp.), *Panonychus* (Yokoyama) (1 sp.), *Petrobia* (Murray) (1 sp.), and *Tetranychus* (Dufour) (20 spp.) [11]. *Tetranychus urticae* Koch and *Oligonychus pratensis* Banks are considered important pests in corn crops because the feeding damage of these mites causes large grain yield losses in corn [7,12]. In California, USA, Bacon et al. [13] reported a reduction in corn yield of up to 32% due to damage caused by *T. urticae*. In Texas, feeding damage of *O. pratensis* can cause economic losses between USD 97.80 and USD 489.00 when silage prices range from USD 10.00 to USD 50.00 per hectare [14].

The Tetranychidae family includes more than 1300 phytophagous species, clustered into 86 genera, divided into 2 subfamilies, Bryobinae and Tetranychinae, distributed throughout the world [11]. Around one hundred species of Tetranychidae are considered pests, and a little more than ten are considered major pests [11,15]. Most of these pests belong to the genera *Tetranychus* Dufour, *Panonychus* Yokoyama, *Oligonychus* Berlese, and *Eutetranychus* Banks [15]. Mites species belonging to these genera are important pests for many crops, fruit trees, vegetables, and ornamentals, and can naturally and temporarily inhabit weeds [15]. The red spider mite, *Tetranychus merganser* Boudreaux (Acari: Tetranychidae), causes severe damage by feeding on different plants crops, such as papaya (*Carica papaya* L.), chili (*Capsicum annuum* L.), and prickle pear cactus (*Opuntia ficus-indica* L.) Miller [11]. In Spider Mites Web for Tetranychidae, *Z. mays* was not found to be a host plant for *T. merganser* [11]. This mite is distributed in China, Mexico, Unites States and Thailand [11]. The damage caused by the feeding of *T. merganser* is severe, destroying the epidermal tissue, the parenchyma, and the chloroplasts of the leaves, which affects the growth, development, and production of the host plant. This damage manifests as white spots near the leaf veins and when the host plant reaches a high population of red spider mite, the spots can merge, causing leaves to turn completely white [16,17].

Before the domestication of plants for agricultural purposes, plants survived and became resistant due to adaptation and natural selection [18]. The effects of resistant plants on pest arthropods can manifest as antibiosis, antixenosis, tolerance, or a combination of these. Antibiosis occurs when the biology of the pest arthropod is negatively affected. Antixenosis occurs when the plant generates anti-feeding and anti-oviposition effects on pest arthropods. Tolerance is a polygenic trait that enables a plant to resist or recover from damage caused by an arthropod pest [18,19]. Plant secondary metabolites, also called allelochemicals, such as alkaloids, flavonoids, terpene lactones, and phenols, affect the health, behavior (antixenosis), growth, or population biology (antibiosis) of an insect or mite [18].

The resistance of different maize inbred lines to *T. urticae* [20,21], *T. cinnabarinus* [22,23], and *O. pratensis* [7,12] have been evaluated. However, there are few studies on the resistance of plants to *T. merganser* [24,25]. Chacón-Hernández et al. [24] evaluated the resistance of three accessions of piquin pepper (*C. annuum* L. var. *glabriusculum* (Dunal) Heiser and Pickersgill) and bean (*Phaseolus vulgaris* L.) to *T. merganser*. Moreover, Treviño-Barbosa et al. [25] evaluated the resistance of seven host plant species (*Thevetia ahouai* (L.) A. DC., *C. papaya*, *P. vulgaris*, *Moringa oleifera* Lam., *Pittosporum tobira* (Thunb.) W.T. Aiton, *Helietta parvifolia* (Gray) Benth., *C. annuum* var. *glabriusculum*) to red spider mite. Furthermore, Ullah et al. [26] and Reyes-Perez et al. [27] evaluated the performance of *T. merganser* at different temperatures. The growth parameters of the population of *T. merganser*, such as the rate of development, survival, reproduction, and longevity are a function of temperature [24,25,26,27]. In a review carried out in the Web of Science database, no report was found on the resistance of *Z. mays* to *T. merganser*, because the red mite is not a pest of the corn crop. However, the red mite has shown potential as an invasive species and has expanded its range of host plants and it is considered a potential pest in Mexican agriculture [28]. This research aimed to assess antibiosis and antixenosis as resistance mechanisms in eleven native maize races cultivated in Tamaulipas, Mexico, to *T. merganser* under laboratory conditions, as well as to obtain information on the chemical composition and morphological characteristics of these maize races, to identify the most resistant maize races for their possible incorporation into breeding programs.

## 2. Results

### 2.1. Qualitative Phytochemical Analysis

The phytochemical analysis of leaf extracts of maize races showed two groups of secondary metabolites: phenols and carotenoids. While saponins, flavonoids, starch, reducing sugars, tannins, and sterols were not detected with their respective tests (Table 1).

### 2.2. Quantitative Phytochemical Analysis

Qualitative analysis is a simple and fast method, but it does not provide a concentration of the bioactive compounds. The phytochemical variables showed differences between the maize races (MANOVA, Wilks test = 0.0043; F = 10.68; *p* = 1.233 × 10^−3^). Table 2 shows the results obtained from the concentrations of total phenols (TPC), flavonoids (TFC), and antioxidant capacity (AC). TFC and AC were significantly higher in the Raton race (0.39 ± 0.01 and 30.73 ± 1.92), respectively, and significantly lower in Nal Tel × Raton (0.14 ± 0.02). Regarding TPC, the Bolita × Raton race (4.09 ± 0.70) presented the highest concentration and the Chalqueño × Tuxpeño race the lowest concentration (2.13 ± 0.68) (Table 3).

### 2.3. Stomata and Leaf Thickness

No trichomes were observed on the third leaf of each maize race. The density and area of stomata and leaf thickness differ significantly between maize races (MANOVA, Wilks test = 0.0017; F = 5.19; *p* = 1.475 × 10^−11^). The Bolita × Raton race had the highest mean number of stomata on the upperside (285.67 ± 45.35) and on the underside (329.23 ± 15.13) of leaves, while the Nal Tel × Raton race presented the lowest average number of stomata (178.67 ± 4.31) per 2 mm^2^. The mean stoma area was higher and lower in the Elotes Occidentales × Tuxpeño (upperside: 1352.83 ± 270.78, underside: 1352.83 ± 270.78) and Tuxpeño (upperside: 654.94 ± 68.74, underside: 654.94 ± 68.74) race, respectively. Meanwhile, the Elotes Occidentales × Tuxpeño race (118.70 ± 1.54) presented the greatest leaf thickness and the Nal Tel × Raton race (104.00 ± 1.39) was the thinnest. (Table 3).

### 2.4. Antixenosis

At 24, 48, and 72 h, the feeding damage and the number of *T. merganser* eggs laid per female differed significantly between maize races (MANOVA, Wilks test = 0.2671; F = 1.96; *p* = 0.0327; Wilks test = 0.2176; F = 2.40; *p* = 0.0084; Wilks test = 0.1929; F = 2.68, *p* = 0.0035). At 24, 48, and 72 h, the number of eggs laid on Celaya race (25.67 ± 17.04, 42.67 ± 26.86, and 49.33 ± 28.54, respectively) was significantly higher than on Raton race (7.00 ± 5.00, 12.67 ± 8.02 and 14.67 ± 9.29, respectively) (LSD’s test, *p* < 0.05). The feeding damage was significantly greater in Celaya race (20.00 ± 0.00, 48.33 ± 29.30, and 65.00 ± 35.00, respectively) than on both Vandeño (11.67 ± 2.89, 21.67 ± 7.64, and 30.00 ± 18.03, respectively) and Raton races (13.33 ± 5.77, 31.67 ± 7.64, and 56.67 ± 20.82, respectively) (Table 4), suggesting antixenosis in the latter two races that reduced feeding by *T. merganser*.

### 2.5. Antibiosis

At 24, 48, and 72 h, the growth rate (ri) of the spider mite did not differ between maize races (MANOVA, Wilks test = 0.3192; F = 0.94; *p* = 0.5615), which suggests no antibiosis to *T. merganser* in these maize races (Table 5).

### 2.6. Mortality

The percentages of red spider mites that survived (live) on leaf squares and were dead (drowned off leaf square) did not differ significantly between maize races (MANOVA, Wilks test = 0.4565; F = 0.61; *p* = 0.9316). At the end of the study, we observed the largest percentage of dead mites was observed on Elotes Occidentales × Tuxpeño (66.67 ± 25.17) and Tuxpeño (63.33 ± 20.82) races, which suggests these maize races are more resistant to *T. merganser* (Table 6).

### 2.7. Principal Component Analysis (PCA)

At 24 h, the principal component analysis (PCA) of variables recorded at 24 h (oviposition, damage, live mites, dead mites, and growth rate (ri)) and total concentration of phenols and flavonoids, antioxidant activity, and the density and area of stomata in the leaves (underside and upperside) of the eleven races of maize, showed own values greater than one in four components. The first four components explain 82.64% of the variance (PC1_24h_ = 35.56, PC2_24h_ = 22.06, PC3_24h_ = 13.57 y PC4_24h_ = 11.45%). We analyzed the first two components because they retained 57.63% of the variation (Table 7).

**Figure 1:**
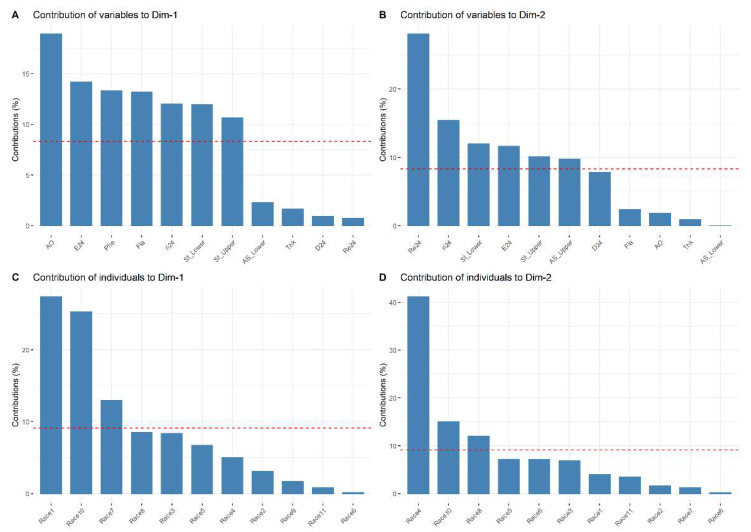
Contribution of the variables (**A**,**B**) and maize races (**C**,**D**) at 24 h. The dotted red line indicates the expected average contribution of the variable and maize race. Variables or maize races with a contribution larger than this cutoff is considered important in contributing to component. Race 1—Raton; Race 2—Olotillo; Race 3—Tuxpeño Norteño; Race 4—Bolita × Raton; Race 5—Elotes Occidentales × Tuxpeño; Race 6—Tabloncillo × Tuxpeño; Race 7—Chalqueño × Tuxpeño; Race 8—Celaya; Race 9—Vandeño; Race 10—Nal Tel × Raton; Race 11—Tuxpeño. E24—average number of eggs laid at 24 h; ri24—growth rate at 24 h; L24—average percentage of live mites at 24 h; Re24—average percentage of dead mites at 24 h; D24—average percentage of feeding damage at 24 h; St_Lower—average density of stomata on the underside of the leaf; St_Upper—average density of stomata on the upperside of the leaf; AS_Lower—area of stomata on the underside of the leaf; AS_Upper—area of stomata in the upperside of the leaf; Fla—total concentration of flavonoids; Phe—total concentration of phenols; AO—antioxidant activity.

Figure 1 shows the most important variables that explain the variability in the data set at 24 h. The variables that contributed the most to PC1_24h_ were antioxidant activity (18.93%); the number of eggs laid (14.20%); the total concentration of phenols (13.34%); total concentration of flavonoids (13.18%); growth rate (11.99%); average density of stomata on the underside of the leaf and average density of stomata on the upperside of the leaf (Figure 1A). The variables that contributed the most to PC2_24h_ were dead mites (28.07%); growth rate (15.40%); average density of stomata on the underside of the leaf (11.99%); the number of eggs laid (11.63%); average density of stomata on the upperside of the leaf (10.11%); and the area of stomata in the upperside of the leaf (9.78%) (Figure 1B). Regarding the maize races, in PC1_24h_, the Raton race contributed 27.37% followed by Nal Tel × Raton (25.27%) and Chalqueño × Tuxpeño (12.93%) (Figure 1C). In PC2_24h_, Bolita × Raton, Nal Tel × Raton, and Celaya contributed 41.17, 15.02, and 11.95%, respectively (Figure 1D).

**Figure 2:**
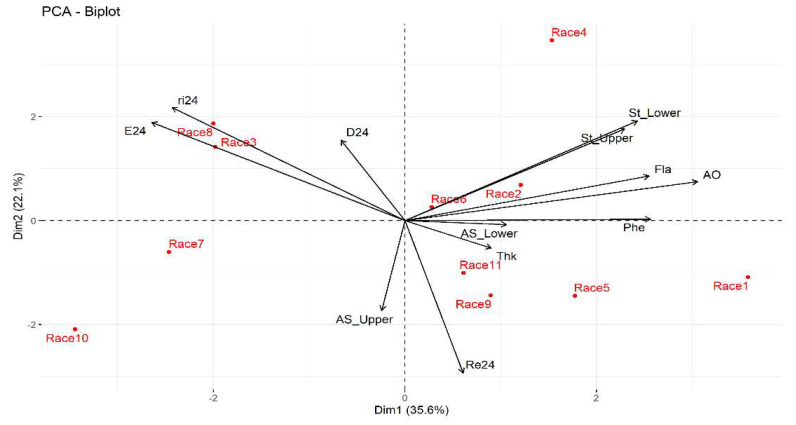
Biplot of the first two principal components of the thirteen variables and 11 maize races at 24 h under laboratory conditions. Race 1—Raton; Race 2—Olotillo; Race 3—Tuxpeño Norteño; Race 4—Bolita × Raton; Race 5—Elotes Occidentales × Tuxpeño; Race 6—Tabloncillo × Tuxpeño; Race 7—Chalqueño × Tuxpeño; Race 8—Celaya; Race 9—Vandeño; Race 10—Nal Tel × Raton; Race 11—Tuxpeño. E24—average number of eggs laid at 24 h; ri24—growth rate at 24 h; L24—average percentage of live mites at 24 h; Re24—average percentage of dead mites at 24 h; D24—average percentage of feeding damage at 24 h; St_Lower—average density of stomata on the underside of the leaf; St_Upper—average density of stomata on the upperside of the leaf; AS_Lower—area of stomata on the underside of the leaf; AS_Upper—area of stomata in the upperside of the leaf; Fla—total concentration of flavonoids; Phe—total concentration of phenols; AO—antioxidant activity.

The dispersion graph (Figure 2) showed that the Tuxpeño Norteño and Celaya races are positively correlated to the variables number of eggs laid, growth rate, and to a lesser extent to the average percentage of feeding damage, which suggests that these maize races are susceptible to *T. merganser*; meanwhile, the same variables are negatively correlated to the Raton race and to a lesser extent with the Elotes Occidentales × Tuxpeño, Vandeño, and Tuxpeño races, which suggests that these maize races present antibiosis (lower population growth of *T. merganser*) and antixenosis (anti-oviposition). The percentage of dead red spider mites was inversely related to the Vandeño race (Figure 2). In addition, the Nal Tel × Raton race has a negative relationship with the density of stomata on the underside and upperside of the leaf, as well as the total concentration of flavonoids and the antioxidant activity present in the leaf.

At 48 h, the PCA showed eigenvalues greater than one in the first four components. The 86.19% of the information (variances) contained in the data were retained by these components. We analyzed the first two components because they retained 61.04% of the variation (Table 8).

**Figure 3:**
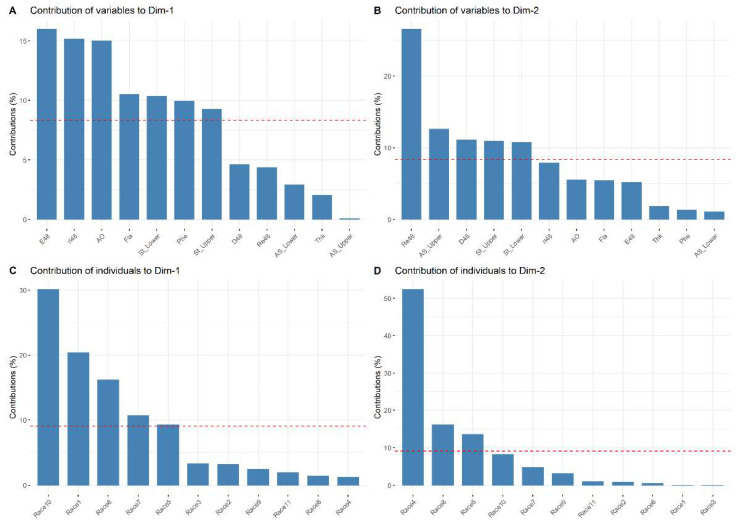
Contribution of the variables (**A**,**B**) and maize races (**C**,**D**) at 48 h. The dotted red line indicates the expected average contribution of the variable and maize race. Variables or maize races with a contribution larger than this cutoff is considered important in contributing to component. Race 1—Raton; Race 2—Olotillo; Race 3—Tuxpeño Norteño; Race 4—Bolita × Raton; Race 5—Elotes Occidentales × Tuxpeño; Race 6—Tabloncillo × Tuxpeño; Race 7—Chalqueño × Tuxpeño; Race 8—Celaya; Race 9—Vandeño; Race 10—Nal Tel × Raton; Race 11—Tuxpeño. E48—average number of eggs laid at 48 h; ri48—growth rate at 48 h; L48—average percentage of live mites at 48 h; Re48—average percentage of dead mites at 48 h; D48—average percentage of feeding damage at 48 h; St_Lower—average density of stomata on the underside of the leaf; St_Upper—average density of stomata on the upperside of the leaf; AS_Lower—area of stomata on the underside of the leaf; AS_Upper—area of stomata in the upperside of the leaf; Fla—total concentration of flavonoids; Phe—total concentration of phenols; AO—antioxidant activity.

The most important variables and maize races that explain the variability in the data set are shown in Figure 3. The variables with the greatest contribution to PC1_48h_ were oviposition (15.97%); ri (15.14%); antioxidant activity (14.99%); the total concentration of flavonoids (10.50%); average density of stomata on the underside of the leaf (10.34%); the total concentration of phenols (9.91%); the average density of stomata on the upperside of the leaf (9.23%) (Figure 3A). The variables with the greatest contribution to PC2_48h_ were dead spider mites (26.51%); stoma area in the upperside leaf (12.58%); percentage of feeding damage (11.10%); the density of stomata on the upperside (10.88%) and underside (10.77%) of the leaves (Figure 3B). Regarding the maize races, the Nal Tel × Raton race contributed 30.04% of the variation for PC1_48h_, followed by Raton (20.35%), Celaya (16.19%), Chalqueño × Tuxpeño (10.67%), and Elotes Occidentales × Tuxpeño (9.22%) (Figure 3C). In PC2_48h_, the races that contributed the most were Bolita × Raton (52.29%), Celaya (16.09%), and Elotes Occidentales × Tuxpeño (13.55%) (Figure 3D).

**Figure 4:**
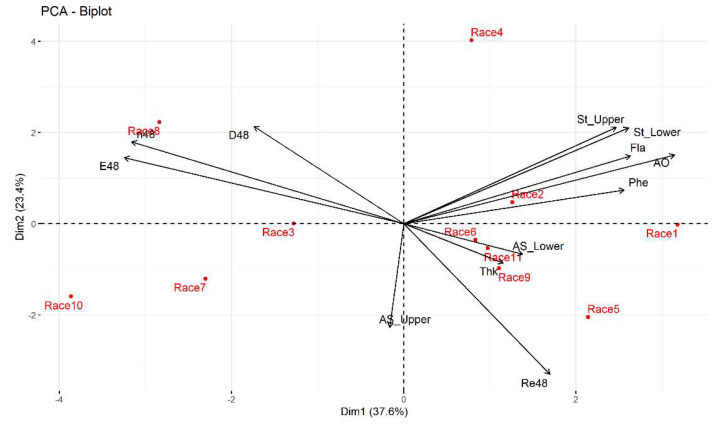
Biplot of the first two principal components of the thirteen variables and 11 maize races at 48 h under laboratory conditions. Race 1—Raton; Race 2—Olotillo; Race 3—Tuxpeño Norteño; Race 4—Bolita × Raton; Race 5—Elotes Occidentales × Tuxpeño; Race 6—Tabloncillo × Tuxpeño; Race 7—Chalqueño × Tuxpeño; Race 8—Celaya; Race 9—Vandeño; Race 10—Nal Tel × Raton; Race 11—Tuxpeño. E48—average number of eggs laid at 48 h; ri48—growth rate at 48 h; L48—average percentage of live mites at 48 h; Re48—average percentage of dead mites at 48 h; D48—average percentage of feeding damage at 48 h; St_Lower—average density of stomata on the underside of the leaf; St_Upper—average density of stomata on the upperside of the leaf; AS_Lower—area of stomata on the underside of the leaf; AS_Upper—area of stomata in the upperside of the leaf; Fla—total concentration of flavonoids; Phe—total concentration of phenols; AO—antioxidant activity.

At 48 h, the dispersion diagram showed that Celaya race had a direct relationship with the variables growth rate and oviposition and to a lesser degree with the percentage of feeding damage, this suggests that Celaya race is susceptible to *T. merganser*. Nal Tel × Raton race showed an inverse relationship with the total flavonoid content, the total phenol concentration, antioxidant activity, and the density of stomata on the underside and upperside of the leaves. The Elotes Occidentales × Tuxpeño race presented a better direct relationship with the number of dead spider mites. The Tuxpeño and Vandeño races both had a direct relationship with the variables thickness of the leaf and the area of the stomata present on the underside of the leaf (Figure 4).

At 72 h, the PCA showed greater eigenvalues than one in the first four components. The 84.74% of the information (variances) contained in the data were retained by these components. We analyzed the first two components because they retained 61.91% of the variation (Table 9).

**Figure 5:**
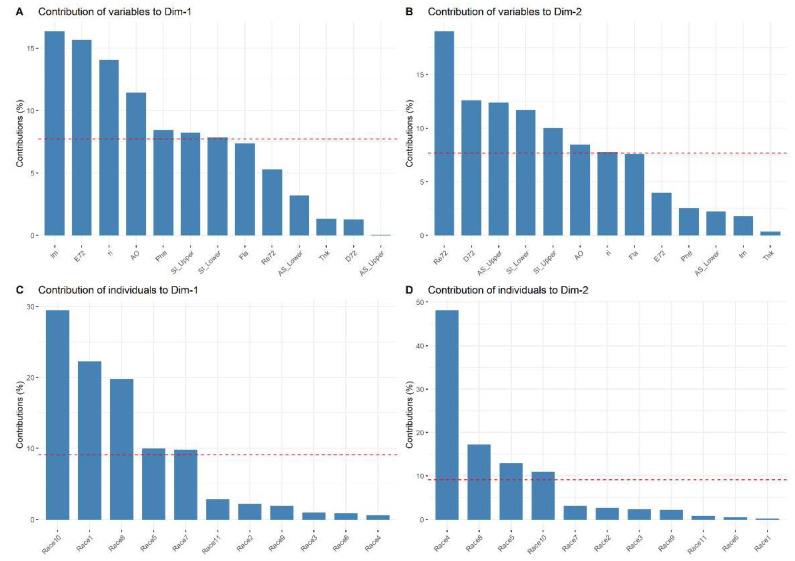
Contribution of the variables (**A**,**B**) and maize races (**C**,**D**) at 72 h. The dotted red line indicates the expected average contribution of the variable and maize race. Variables or maize races with a contribution larger than this cutoff is considered important in contributing to component. Race 1—Raton; Race 2—Olotillo; Race 3—Tuxpeño Norteño; Race 4—Bolita × Raton; Race 5—Elotes Occidentales × Tuxpeño; Race 6—Tabloncillo × Tuxpeño; Race 7—Chalqueño × Tuxpeño; Race 8—Celaya; Race 9—Vandeño; Race 10—Nal Tel × Raton; Race 11—Tuxpeño. E72—average number of eggs laid at 72 h; ri72—growth rate at 72 h; L72—average percentage of live mites at 72 h; Re72—average percentage of dead mites at 72 h; D72—average percentage of feeding damage at 72 h; Im—number of immature mites; St_Lower—average density of stomata on the underside of the leaf; St_Upper—average density of stomata on the upperside of the leaf; AS_Lower—area of stomata on the underside of the leaf; AS_Upper—area of stomata in the upperside of the leaf; Fla—total concentration of flavonoids; Phe—total concentration of phenols; AO—antioxidant activity.

Figure 5 shows the variables and maize races with the highest contribution for the variability in PCA made at 72 h were the number immature (16.29%); oviposition (16.60%); ri (13.99%); antioxidant activity (11.41%); total concentration of phenols (8.41%); the density of stomata on the upperside (8.18%) and underside (7.79%) (Figure 5A). The variables and maize races with the highest contribution for PC2_72h_ were dead spider mites (18.98%); percentage of feeding damage (12.56%); area of the stomata present on the upperside of the leaf (12.36%); density of stomata on the underside (11.66) and upperside (9.98%) of the leaves; antioxidant activity (8.44%); ri (7.75%) (Figure 5B). Regarding the maize races, those with the most contribution to PC1_72h_ were Nal Tel × Raton (29.42%), Raton (22.21%), Celaya (19.73%), Elotes Occidentales × Tuxpeño (9.94%), and Chalqueño × Tuxpeño (9.74%) (Figure 5C). In PC2_72h_, the races Bolita × Raton (48.08%), Celaya (17.10%), Elotes Occidentales × Tuxpeño (12.79%), and Nal Tel × Ratón (10.78%) were the most important (Figure 5D).

**Figure 6:**
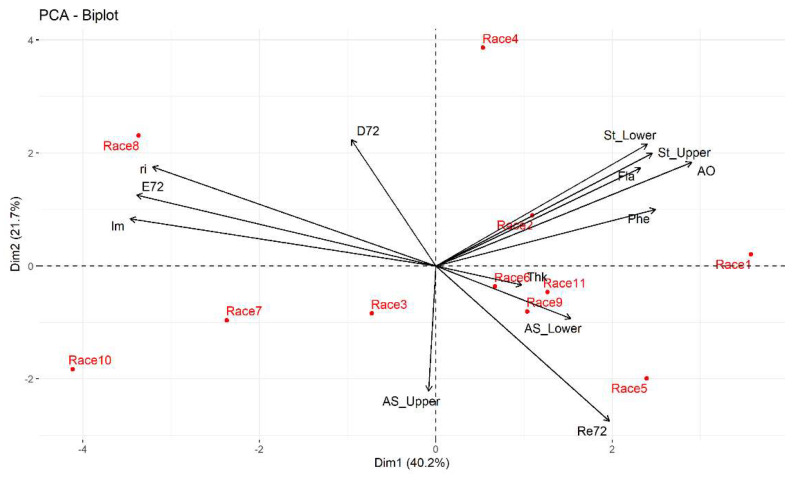
Biplot of the first two principal components of the thirteen variables and 11 maize races at 72 h under laboratory conditions. Race 1—Raton; Race 2—Olotillo; Race 3—Tuxpeño Norteño; Race 4—Bolita × Raton; Race 5—Elotes Occidentales × Tuxpeño; Race 6—Tabloncillo × Tuxpeño; Race 7—Chalqueño × Tuxpeño; Race 8—Celaya; Race 9—Vandeño; Race 10—Nal Tel × Raton; Race 11—Tuxpeño. E72—average number of eggs laid at 72 h; ri72—growth rate at 72 h; L72—average percentage of live mites at 72 h; Re72—average percentage of dead mites at 72 h; D72—average percentage of feeding damage at 72 h; Im—number of immature mites; St_Lower—average density of stomata on the underside of the leaf; St_Upper—average density of stomata on the upperside of the leaf; AS_Lower—area of stomata on the underside of the leaf; AS_Upper—area of stomata in the upperside of the leaf; Fla—total concentration of flavonoids; Phe—total concentration of phenols; AO—antioxidant activity.

The dispersion graph (Figure 6) shows that the Celaya race is strongly related to the variables growth rate, oviposition, and immature mites, this suggests that the Celaya race was most susceptible to *T. merganser*, while the Elotes Occidentales × Tuxpeño race is positively related to the variable dead spider mites and has a negative relationship with the variables growth rate (antibiosis), oviposition (antixenosis), and to a lesser extent with the variable damage by mite feeding (antixenosis), this suggests Elotes Occidentales × Tuxpeño race was most resistant to *T. merganser*.

## 3. Discussion

Phytophagous arthropods identify their host plant as a food source, but the quality and quantity of the food source are important factors affecting the longevity and fecundity of herbivorous arthropods [29,30]. Our results showed that maize races affected the behavior (antixenosis) and biology (antibiosis) of *T. merganser*. At each observation time (24, 48, and 72 h), the feeding, oviposition, and fecundity of the red spider mite were affected by the different races of maize. The results of antixenosis at observation times show that Elotes Occidentales × Tuxpeño, Tuxpeño, and Vendeño races reduced the oviposition and feeding of *T. merganser*, while oviposition and feeding by red spider mite increased on the Celaya race. The non-preference of the red spider mite for some races of maize could be due to differences in the total concentration of flavonoids (Table 2), the thickness of the leaf, and the number of stomata present in the leaf (Table 3). Secondary metabolites such as flavonoids can deter oviposition and feeding of a phytophagous arthropod [18,31,32]. The thickness of the leaf can be a physical barrier so that mites such as *Tetranychus urticae* Koch and *T. lintearius* Dofour cannot feed on *Phaseolus vulgaris* L. (Fabaceae) and *Ulex europaeus* L. (Leguminosae), respectively [33,34]; therefore, these spider mites insert their stylet into the stomata so they can feed on their host plants [34].

The literature does not refer to other studies on the resistance of maize races to *Tetranychus* spp. However, other studies have evaluated the antibiosis and antixenosis of maize inbred lines to *Tetranychus* spp. and *Oligonychus* sp. and their findings are very similar to ours. Additionally, oviposition, growth rate, and feeding rate differ between maize races. Mite fecundity is affected by plant secondary metabolites, leaf nutrition, leaf age, and leaf surface structure [35]. Tadmor [23] reported differences in the number of eggs laid per day per female of *T. cinnabarinus* on ten inbred maize lines. They documented performance from 0.2 to 1.4 E/F/D in four days. While Paoulo et al. [36] found no difference in the number of eggs laid by *T. urticae* on maize plants reinfested with the spider mite compared to plants with mites. They concluded that there is a direct resistance induction of *Z. mays* to *T. urticae*. Bui et al. [7] evaluated the resistance of 38 inbred maize lines to *T. urticae* and *O. pratensis*. They found that *T. urticae* (median ~8–50) exhibited greater variation than *O. pratensis* (median ~30–50) when compared with progeny per female (eggs and viable mites, all stages) in six days of evaluation under greenhouse conditions; meanwhile, Bui et al. [12] evaluated inbred maize W22, from which Ds transposon insertions were recovered in three genes responsible for DIMBOA-Glc synthesis—BX1, BX2, and BX6. They found that the number of progeny per female of *T. urticae* and *O. pratensis* on the inbred maize line (M22) and on the mutants maize (bx1::Ds, bx2::Ds, and bx6::Ds) were different and similar, respectively.

Other studies have reported that the number of eggs laid by *T. merganser* is related to the host plant [24,25]. Chacón-Hernández et al. [24] reported lower oviposition of *T. merganser* female (66.00 ± 15.31, 66.33 ± 8.67 and 44.33 ± 21.40) on three accessions (BGH-425, BGH-426 and Ch1) of *C. annuum* var. *glabriusculum* than on *P. vulgaris* (166.00 ± 7.23) at 120 h, under laboratory conditions at 27 ± 2 °C, 60–70% RH and a photoperiod of 12:12 L:D; meanwhile, Treviño-Barbosa et al. [25] reported that the daily oviposition rate of the red spider mite was higher in *C. papaya* (7.46 ± 0.18 eggs/female/day) than on *P. vulgaris* (4.41 ± 0.22), *M. oleifera* (3.57 ± 0.12), *C. annuum* var. *glabriusculum* (2.92 ± 0.12), *H. parvifolia* (1.49 ± 0.04), *P. tobira* (1.10 ± 0.09), and *T. ahouai* (0.97 ± 0.09) at 28 ± 1 °C, 70–80% relative humidity and photoperiod of 12:12 L:D. These variations could be attributed to differences in plant species and cultivar types, as they may have differences in nutrient content, morphological characteristics and in secondary metabolite levels, as well as environmental factors [26,35,37,38,39].

Resistant plants cause mites not to feed, causing them to be thin and weak, or if they do feed, they cannot digest the food material, causing them to swell and turn black [40]. In the Tabloncillo × Tuxpeño and Vandeño races, feed intake of *T. merganser* was lower compared with other maize races (Table 4) and by visual observations, we found that the red spider mites were thinner in these maize races than in the others. This may be due to the differences in the morphological characteristics and secondary metabolites. Secondary metabolites such as flavonoids and phenols that were found stored in the cell walls of leaves deter feeding and oviposition of arthropods [18]. Hence, the maize races differ in their suitability as possible hosts for red spider mites when measured in terms of fecundity and as resources for feeding. In this regard, Ullah et al. [40] documented that *T. urticae* and *T. kanzawai* cause different feeding damage rates in 23 cultivars and 20 lines of cucumber (*Cucumis sativus* L.). Bynum et al. [14] reported that *T. urticae* caused different feeding damage rates in 12 maize inbred lines. Besides, Bui et al. [7] documented that *T. urticae* causes the highest feeding damage rate on maize inbred line B73 than *O. pratensis*. Treviño-Barbosa [25] reported a higher feeding damage of *T. merganser* in *C. papaya* (47.00 ± 0.73%) than to *P. vulgaris* (40.67 ± 1.76%), *M. oleifera* (32.33 ± 1.76%), *C. annuum* var. *glabriusculum* (30.67 ± 1.20%), *H. parvifolia* (27.00 ± 2.52%), *P. tobira* (26.33 ± 3.18%), and *T. ahouai* (24.33 ± 0.67%) at 28 ± 1 °C, 70–80% relative humidity, and photoperiod of 12:12 L:D.

In this research work, the antibiosis of the different maize races was indicated by the growth rate (ri). Although the MANOVA did not show significant differences in the growth rate of the red spider mite between the maize races, the PCA showed a strong inverse relationship between the ri and the Elotes Occidentales × Tuxpeño race and to a lesser extent with the Tuxpeño and Vandeño in the different observation times (see Figure 2, Figure 4 and Figure 6). Factors such as survival and fertility rate affect ri; hence, ri adequately summarizes the physiological qualities of an insect with its ability to increase its population [41]. In addition, this parameter is the most appropriate for evaluating plants resistance to herbivorous arthropods. The PCA showed that the immature mites, oviposition, and growth rate of *T. merganser* were inversely related with the Elotes Occidentales × Tuxpeño, Vandeño and Tuxpeño races. In addition, these maize races did not present differences in the total phenols concentration. This bioactive compound present in maize plants may be an antibiosis factor for herbivorous arthropods, such as *Sitophilus zeamais* Motschulsky (Coleoptera: Curculionidae), *Prostephanus truncatus* (Horn) (Coleoptera: Bostrichidae) [42], and *Sesamia nonagrioides* Lefèbvre (Lepidoptera: Noctuidae) [43,44]. On the other hand, Agut et al. [45] found that flavonoid compounds such as p-coumaric acid present in *Citrus aurantium* (L.) caused resistance to *T. urticae*. This secondary metabolite participates in the biosynthesis of lignin polymers, resulting in the formation of physical barriers to reduce the palatability of the plant [46]. Native maize races, such as the Tuxpeño race, contain high contents of p-coumaric acid [47].

Cultivars that support low population densities of herbivorous arthropods are an important part of pest management [48]. To conclude, this research showed that *T. merganser* feeds and develops in the eleven native maize populations, but also showed that the Elotes Occidentales × Tuxpeño, Tuxpeño, and Vandeño races are resistant to red spider mite. This resistance is due to their morphological characteristics and the secondary metabolites present in their leaves. Further, research is necessary on the behavior and biology of *T. merganser* under field conditions. Such research might give us a clearer idea of the effects of the red spider mite on the yield of the maize races.

## 4. Materials and Methods

### 4.1. Red Spider Mite Colony

Our colony of red spider mite was started with biological material obtained from the Population Ecology Laboratory at the Institute of Applied Ecology at the Autonomous University of Tamaulipas. The collected mites were placed on bean plants (*Phaseolus vulgaris* var. Michoacan) under greenhouse conditions at 30 ± 2 °C and 70 ± 10% relative humidity (RH).

### 4.2. Plant Material

We selected eleven native maize populations, each one representative of each race of maize grown in the state of Tamaulipas, Mexico [3,49,50]. The seeds were provided by the Germplasm Bank of the Institute of Applied Ecology, Autonomous University of Tamaulipas (Table 10).

### 4.3. Preparation of Maize Plants and Screening for Resistance

The germination of the eleven native maize populations was carried out under greenhouse conditions (30 ± 2 °C and 70 ± 10% RH). We used polyethylene bags (5 × 7 × 10 cm high) with a growth medium of soil:vermiculite (1:0.5), irrigated only with water, to avoid possible effects of macroelements on the red spider mite [51,52].

We used the modified third-leaf technique of Tadmor et al. [23]. Four seeds of each maize race were sown per bag and repeated three times. When the seedlings showed the third leaf grown (between 15 and 20 cm in length), three leaves were cut and immediately transported to the Population Ecology Laboratory to carry out the bioassays.

### 4.4. Phytochemical Extract Analysis

#### 4.4.1. Preparation of Extracts

Samples were dried at 45 °C during 96 h and then pulverized in a mortar. The extraction was carried out by maceration of sample in absolute methanol in a one: four ratio (*w*/*v*) during 30 min at room temperature (28–30 °C), followed by centrifugation at 8000× *g* for 10 min. The clarified supernatant was used as a source of phytochemicals for subsequent analyses.

#### 4.4.2. Total Phenolic Compounds (TPC)

The TPC contents were quantified with the Folin–Ciocalteu reagent according to Singleton et al. [53]. Volumes of 250 μL of each sample or standards were mixed with 125 μL of 1 N Folin–Ciocalteu reagent (Sigma-Aldrich, St. Louis, MO, USA) and followed by incubation during 5 min. After, 625 μL of 20% Na_2_CO_3_ (CTR, Monterrey, Nuevo Leon, Mexico) were added and mixtures were incubated in darkness for 2 h. Then, the absorbance of each reaction was recorded at 750 nm (UV-6000, Metash instruments Co., Ltd., Shanghai, China). The concentration of the samples was determined according to a standard curve prepared with a 0.1 mg/mL solution of gallic acid (Sigma-Aldrich, St. Louis, MO, USA) in a range of 1–8 μg/mL. Concentrations were expressed in mg gallic acid equivalents per gram of dry weight.

#### 4.4.3. Determination of Total Flavonoids

The concentration of flavonoids was calculated according to the method suggested by Chang et al. [54], mixing 1 mL of sample, 1.5 mL of ethanol (95%) (CTR, Monterrey, Nuevo Leon, Mexico), 0.1 mL of 10% aluminum chloride (Sigma-Aldrich, St. Louis, MO, USA), 0.1 mL of 1M potassium acetate (Sigma-Aldrich, St. Louis, MO, USA), and 2.8 mL of sterile distilled water. The mixtures were incubated during 40 min at room temperature. Absorbance obtained in each reaction was recorded at 415 nm and used for determination according to a standard curve, which was prepared with quercetin as a standard (Sigma-Aldrich, St. Louis, MO, USA) at concentrations of 10–100 μg/mL. The recorded concentrations were expressed in mg quercetin equivalents per gram of dry weight.

#### 4.4.4. Antioxidant Capacity Detection Using FRAP

The capacity to reduce ferric ions was determined with an adapted version of the method proposed by Benzie and Strain [55]. An aliquot (10 µL) of extract was added to 200 µL of FRAP reagent (10 parts of 300 mM sodium acetate buffer at pH 3.6, 1 part of 10 mM TPTZ solution, and 1 part of 20 mM FeCl_3_·6H_2_O solution) and the reaction mixture was incubated at 37 °C. The increase in absorbance at 595 nm was measured after 60 min. The antioxidant capacity of the extracts was expressed as mM Trolox equivalents per gram of dry weight. The standard curve was generated with Trolox in a range of 100–1200 μM. Detections were carried out in triplicate.

#### 4.4.5. Detection of Phenolic Compounds with KMnO_4_

To 100 μL of sample, 100 μL of distilled water was added. Detection was performed by adding 30 μL of 0.1% KMnO_4_ (CTR, Monterrey, Nuevo León, Mexico). The reaction was considered positive when the change in purple color was yellowish or greenish. As in all other reactions, distilled water, 50% ethanol, and absolute ethanol were used as control [56].

#### 4.4.6. Detection of Polyphenols with FeCl_3_

A measure of 100 μL of distilled water were added to 100 μL of each sample and the reactions were carried out with the addition of 30 μL of 0.5% FeCl_3_ (CTR, Monterrey, Nuevo Leon, Mexico). The reaction was positive when dark green, blue–dark blue, or even blackish tones appeared in the mixture [56].

#### 4.4.7. Detection of Sterols by Liebermann–Burchard Reaction

For each extract, a 300 μL aliquot was evaporated into a test tube and then resuspended in 300 μL of chloroform. In addition, a reaction solution was prepared by mixing 1 mL of acetic anhydride (CTR, Monterrey, Nuevo León, Mexico), with 1 mL of concentrated chloroform and three drops of concentrated sulfuric acid, which were mixed by stirring. For the development of the reaction, 50 μL of the reaction solution in the tube were mixed with the 300 μL of the extract resuspended in the chloroform, this mixture was left to rest for 20–25 min, and it was considered as positive when a red-violet-colored area appeared [56].

#### 4.4.8. Detection of Alkaloids by Wagner Reaction

The samples of 75 μL of each extract were heated to 95 °C and 75 μL of HCl (10%) was added (CTR, Monterrey, Nuevo Leon, Mexico). The tubes were left at room temperature to cool them and then 20 μL of Wagner reagent was added and mixed. The reaction was considered positive when a garnet precipitate formed [56].

#### 4.4.9. Flavonoid Detection by Shinoda Reaction

For each extract, at a 100 μL aliquot, some metallic magnesium fragments and three drops of 25% HCl solution were added. A positive reaction was considered when the color of the solution changed to pink, yellow, or orange [56].

#### 4.4.10. Tannin Detection

The detection was based on the precipitation of gelatin in saline solution. For each extract, ten tubes were prepared with 2 mL of one of a solution, as indicated: three tubes with a 10% NaCl solution, three tubes with 2% gelatin solution dissolved in 10% NaCl, and three with a solution of 2% gelatin in distilled water, and one tube was left as a control using the solvent from the corresponding extraction. Subsequently, 500 μL of the extract were added to each tube and allowed to stand for 10 min. During this time, the tubes were observed. The reaction was positive when a whitish precipitation appeared or the formation of clots was observed in the tubes with gelatin and gelatin with 10% NaCl, but not in the control of distilled water nor the tube with 10% NaCl alone [56].

#### 4.4.11. Detection of Saponins

Measures of 300 μL of extract and 300 μL of boiling water were mixed in a tube, then cooled and vigorously shaken during 30 s. Foaming for more than 10 min was considered a positive reaction for the presence of saponin-like compounds [56].

#### 4.4.12. Detection of Starch

The detection of starch was carried out by mixing 2 mL of sample with 1–2 drops of Lugol’s iodine solution, which was prepared by dissolving 10 g KI in about 20–30 mL of distilled water, then adding 5 g of iodine and heating it gently with constant mixing until iodine was dissolved. Then, volume was completed to 100 mL adding distilled water. Starch detection was considered positive when sample changed from brown to blue-black or purple. A 2% solution of starch from corn in water was used as positive control [56].

#### 4.4.13. Detection of Reducing Sugars by Fehling Test

First, a Fehling solution was prepared. Solution A was carried out by dissolving 7 g of CuSO_4_·7H_2_O in 100 mL of water. Solution B was prepared by dissolving 24 g of KOH and 34.6 g of potassium sodium tartrate in 100 mL water. Both solutions were mixed in equal volumes just before using. Two drops were applied to 1 mL of sample and then stirred during 10 s and then the test mixes were processed in a boiling water bath for 2 min. The appearance of a reddish-brown precipitate was considered as a positive result for reducing sugars. A solution of 0.5% of glucose in water was used as a positive control [56].

#### 4.4.14. Detection of Carotenoids

Reaction was based on detection with sulfuric acid reaction [57]. To 1 mL of sample, 1–2 drops of concentrated sulfuric acid were added and mixed well. Blue tones showed the solution was positive for carotenoids.

### 4.5. Morphological Characteristics

#### 4.5.1. Density and Area of Stomata

We quantified the number of stomata for the adaxial and abaxial surface of the third leaf, using leaf impressions and optical microscopy. For the impressions, two layers of transparent enamel were applied on the adaxial and abaxial surfaces, allowing 15 min to dry between each coat. Subsequently, the enamel film was peeled off and fixed on a slide. The samples were observed and photographed with a binocular microscope (UNICO M280, UNICO, Dayton, NJ, USA) equipped with a MiniVID 5 mp LWScientific digital camera. Stomatal density was determined by 1 mm^2^ quadrant using the 10× objective. For the measurement of the width and length of the stomata, the 40× objective was used. Measurement of lamella thickness was carried out with cross-sections, using a high-precision ocular micrometer (1 mm/100 div.) (Muhwa eCommerce Co., Zhi Ying M806301, Shanghai, China). Determinations were performed in triplicate for each race of maize.

#### 4.5.2. Leaf Thickness

We made three cuts at different heights of the leaf: (1) near the apex, (2) in the middle, and (3) at the base of the leaf. Immediately, transverse cuts were made manually with the help of a stainless-steel razor blade (Super-Max platinum, Vidyut Metallics, Thane, India). To measure the thickness, the measurement of the external surface of the adaxial epidermis to the external surface of the abaxial epidermis was considered. The measurement was made with the help of a binocular microscope (UNICO M280, UNICO, Dayton, NJ, USA) and with the Toupview software. All measurements were performed in triplicate for each race of maize.

### 4.6. Experimental Design

We used the modified sand technique of Ahmadi [58]. Three squares of 2 × 2 cm were cut from the leaves of each native maize plant. The underside of the leaf square was placed upwards on cotton saturated with water and placed in a Petri dish of 5 cm diameter. Twenty adult of *T. merganser* females and ten males were placed on each leaf maize square to enhance reproduction and oviposition. After 12 h, the males were removed, leaving only ten females to the assay. The experiment was carried out under laboratory conditions at 27 ± 1 °C, 70–80% RH, and photoperiod 12:12 L:D (Light: Dark). We randomly assigned three leaf squares to eleven groups, one group for each maize race. The leaf squares of each maize race were the replicas, and had 3 replicas per group, with 33 in total. Adult female red spider mites were two days old when transferred to the leaf square.

#### 4.6.1. Antixenosis

Antixenosis was determined under two criteria. First, non-oviposition preference of *T. merganser* over any race of maize. We recorded the number of eggs laid at 24, 48, and 72 h after infestation. Previous studies with the same experimental conditions have shown that the red spider has high rates of oviposition and food intake on bean disks and papaya [24,25]. Second, the damage index was used to measure non-preference for feeding, which was estimated visually on each leaf square using an ordinal scale given by Hussey and Parr [59] and Nachman and Zemek [60] and converted to percentages, where 0 = 0% damage (no feeding damage); 1 = 1–20% incipient damage, one or two small feeding patches; 2 = 21–40% feeding patches tending to coalesce, but only 2/3 of leaf square affected; 3 = 41–60% 2/3 of leaf square with feeding marks as chlorotic patches; 4 = 61–80% dense feeding marks over entire leaf square but appearance still green; 5 = 81–100% feeding damage (a dense mark or wilt caused by *T. merganser* feeding of the leaf square). The percentage of damage was recorded at 24, 48, and 72 h after infestation.

#### 4.6.2. Mortality

We used the Chacón-Hernández et al. [24] formula to measure the percentage of mortality of *T. merganser*. Mortality was measured by the average percent of dead individuals (drowned) outside the leaf square (Σd_i_/*n*) × 100, where di is the number of drowned individuals and *n* is the number of individuals on the leaf square. The number of dead and alive mites was recorded at 24, 48, 72, and 96 h.

Antibiosis: We used the growth rate (ri), as a parameter to determine the antibiosis on the population of *T. merganser*. ri was calculated with the following equation [61]:

ri = [ln(N_t_/N_0_)] × (1/t) (1)

where N_t_ is the number of individuals at time t (surviving adult females plus the eggs laid at the end of the bioassay), N_0_ is the number of individuals at time 0 (initial cohort = 10 adult females of *T. merganser*), and t is the number of days elapsed from the start to the end of the bioassay (equal to 3 days). Positive values of ri indicate a growing population, negative values indicate a declining population, and ri = 0 indicates a stable population [62].

### 4.7. Statistic Analysis

The number of laid eggs, dead mites, live mites, and the percentage of damage by feeding were registered at 24, 48, and 72 h. Growth rate was calculated on the third day after infestation and immature mite counts were recorded on the seventh day. All counts were performed with the aid of a dissecting microscope (UNICO Stereo and Zoom Microscopes ZM180, Dayton, NJ, USA). The means and standard deviation (SD) of each variable were calculated.

We used multivariate analysis of variance (MANOVA) to test differences between maize races in terms of phytochemical (phenols, flavonoids, and antioxidant activity), morphological parameters (leaf thickness, stomata density, and stomata area), and the biological and physiological characteristics of *T. merganser* (eggs number, immature, alive, dead, food intake). Additionally, we used multiple pairwise comparisons (LSD test, *p* ≤ 0.05) to separate maize races according to these parameters.

We used the average of each variable to apply a principal components analysis (PCA), this described the variation of the variables and that the first components represent a substantial proportion of the variation in the original variables [63], which showed the convenient lower dimensions of each variable that were useful to analyze the resistance mechanisms of the eleven maize races. We used the eigenvalues and the cumulative percent variance to determine the number of principal components [64,65]. We completed three PCAs, one for each recording time (24, 48, and 72 h), to better analyze the behavior of *T. merganser* on the eleven maize races.

The contribution of each variable and maize race was determined by the Kassambara [65] criterion, which indicates that, if the contribution is uniform, then the expected value would be C = 1/length (variables or maize races). For 24 and 48 h, 12 variables were used, and for 72 h, 13 variables were used; therefore, C = (1/12) × 100 = 8.33% and C = (1/13) × 100 = 7.69%, respectively. In the case of the contribution of the maize races: C = (1/11) × 100 = 9.10%. The total contribution of a given variable, when explaining the variations retained by two principal components, PC1 and PC2, was calculated as, contrib = [(C1 × Eig1) + (C2 × Eig2)/(Eig1 + Eig2)]. Where C1 and C2 are the contributions of the variable in PC1 and PC2, respectively, Eig1 and Eig2 are the eigenvalues of PC1 and PC2, respectively. Therefore, for a given component, in 24 or 48 h, a variable with a contribution greater than 8.33% and for 72 h greater than 7.69% is considered the important variable in the contribution to the principal component. In the three PCAs, the phytochemical variables and morphological characteristics were included, to determine were these variables showed any relationship with mortality, survival, feeding damage, number of eggs laid, and growth rate of *T. merganser* in different observation times (24, 48, and 72 h). For this analysis, the FactoMineR package was used, as well as for the factoextra graphs (visualization based on ggplot2) [65]. All analyses were performed in R software version 4.1.0 [66].

## Figures and Tables

**Table 1:** Secondary metabolites (“+” = present; “−” = absent) in methanol extract of races of maize.

Races	Sap.	Alk.	Phe.	Phen.	Flav.	Sol.	Free	Tan.	Cara.	St.
Raton	−	−	−	+	−	−	−	−	+	−
Olotillo	−	−	−	+	−	−	−	−	+	−
Tuxpeño Norteño	−	−	−	+	−	−	−	−	+	−
Bolita × Raton	−	−	−	+	−	−	−	−	+	−
Elotes Occidentales × Tuxpeño	−	−	−	+	−	−	−	−	+	−
Tabloncillo × Tuxpeño	−	−	−	+	−	−	−	−	+	−
Chalqueño × Tuxpeño	−	−	−	−	−	−	−	−	+	−
Celaya	−	−	−	+	−	−	−	−	+	−
Vandeño	−	−	−	++	−	−	−	−	+	−
Nal Tel × Raton	−	−	−	−	−	−	−	−	+	−
Tuxpeño	−	−	−	+	−	−	−	−	+	−

Signs are negative (−), positive (+), intense positive (++) of observed response. Sap.—saponins (Foam test); Alk.—alkaloids (Draggendorff test); Phe.—phenolics (FeCl_3_ test); Phen.—phenolics (KMnO₄ test); Flav.—flavonoids (Shinoda’s test); Sol.—soluble starch (KOH and H_2_SO_4_ test); Free—free reducing sugars (Fehling test); Tannins (FeCl_3_ test); Cara.—carotenoids (H_2_SO_4_ test); St.—sterols (Burchard’s test).

**Table 2:** Total phenols concentration (TPC), total flavonoids concentration (TFC), and antioxidant capacity in the leaf of different races of maize.

Races	Total Flavonoids (mg QE/g) *	Total Phenols (mg GAE/g)	Antioxidant Activity (mM ET/g)
Raton	0.39 ± 0.01 ^a^	3.07 ± 0.77 ^b,c,d^	30.73 ± 1.92 ^a^
Olotillo	0.25 ± 0.01 ^b^	3.78 ± 0.43 ^a,b^	23.99 ± 1.61 ^b,c^
Tuxpeño Norteño	0.20 ± 0.01 ^c^	2.30 ± 0.13 ^d^	16.55 ± 1.50 ^d^
Bolita × Raton	0.23 ± 0.02 ^b^	4.09 ± 0.70 ^a^	27.67 ± 2.76 ^a,b^
Elotes Occidentales × Tuxpeño	0.23 ± 0.02 ^b^	3.44 ± 0.47 ^a,b,c^	25.61 ± 1.62 ^b,c^
Tabloncillo × Tuxpeño	0.18 ± 0.01 ^c^	3.00 ± 0.24 ^b,c,d^	24.22 ± 1.98 ^b,c^
Chalqueño × Tuxpeño	0.19 ± 0.01 ^c^	2.13 ± 0.68 ^d^	17.89 ± 3.10 ^d^
Celaya	0.23 ± 0.02 ^b^	2.71 ± 0.41 ^c,d^	24.27 ± 1.21 ^b,c^
Vandeño	0.25 ± 0.02 ^b^	3.80 ± 0.32 ^a,b^	25.34 ± 1.75 ^b,c^
Nal Tel × Raton	0.14 ± 0.02 ^d^	2.34 ± 1.23 ^d^	12.10 ± 1.03 ^e^
Tuxpeño	0.19 ± 0.01 ^c^	2.98 ± 0.15 ^b,c,d^	23.69 ± 4.37 ^c^

* Means values and ± standard deviation (SD) is presented. Different letters indicate significant differences (*p* < 0.05; MANOVA and LSD’s test).

**Table 3:** Mean number of stomata (density) per leaf and stoma area, and leaf thickness of eleven races of maize.

Race	Stomata Density (for Each 2 mm^2^) per Leaf *		Stoma Area (µm^2^)		Leaf Thickness (µm)
	Upper	Lower	Upper	Lower	
Raton	272.00 ± 23.27 ^a,b^	291.54 ± 26.32 ^b,c,d,e^	1173.11 ± 276.21 ^a,b,c^	1177.59 ± 228.07 ^a,b,c^	109.2 ± 1.48 ^d,e^
Olotillo	229.33 ± 46.18 ^b,c^	316.44 ± 11.10 ^a,b^	1104.34 ± 139.12 ^a,b,c,d,e^	1107.44 ± 249.07 ^a,b,c^	118.00 ± 1.67 ^a,b^
Tuxpeño Norteño	245.34 ± 29.68 ^a,b,c^	277.33 ± 18.47 ^c,d,e,f^	1044.11 ± 215.55 ^b,c,d,e^	1334.37 ± 560.27 ^a,b^	105.7 ± 1.47 ^f,g^
Bolita × Raton	285.67 ± 45.35 ^a^	329.23 ± 15.13 ^a^	820.42 ± 15.79 ^e,f^	1120.90 ± 45.24 ^a,b,c^	107.00 ± 1.65 ^e,f^
Elotes Occidentales × Tuxpeño	217.00 ± 40.03 ^c,d^	298.77 ± 43.18 ^a,d,c,d^	1352.83 ± 270.78 ^a^	1430.98 ± 313.81 ^a^	118.70 ± 1.54 ^a^
Tabloncillo × Tuxpeño	237.31 ± 8.01 ^b,c^	304.00 ± 4.70 ^a,b,c^	1027.52 ± 126.87 ^c,d,e^	975.7 ± 68.11 ^b,c^	111.00 ± 1.39 ^d^
Chalqueño × Tuxpeño	208.67 ± 10.72 ^c,d^	248.00 ± 7.85 ^f,g^	1131.20 ± 137.87 ^a,b,c,d^	923056 ± 89.90 ^b,c^	116.00 ± 1.35 ^b,c^
Celaya	203.30 ± 3.99 ^c,d^	272.03 ± 6.95 ^d,e,f,g^	860.39 ± 130.69 ^d,e,f^	891.11 ± 78.21 ^b,c^	114.20 ± 1.20 ^c^
Vandeño	208.37 ± 2.38 ^c,d^	266.41 ± 10.72 ^e,f,g^	988.67 ± 42.97 ^c,d,e^	1075.01 ± 196.48 ^a,b,c^	114.70 ± 1.39 ^c^
Nal Tel × Raton	178.67 ± 4.31 ^d^	242.70 ± 5.76 ^g^	1312.42 ± 190.74 ^a,b^	1054.92 ± 35.73 ^a,b,c^	104.00 ± 1.39 ^g^
Tuxpeño	238.30 ± 21.34 ^b,c^	280.63 ± 13.34 ^c,d,e^	654.94 ± 68.74 ^f^	834.33 ± 431.28 ^c^	110.70 ± 1.73 ^d^

* Means values and ± standard deviation (SD) is presented. Different letters indicate significant differences (*p* < 0.05; MANOVA and LSD’s test).

**Table 4:** Daily (hours = h) mean number of eggs laid and percentage of feeding damage of *Tetranychus merganser* in 11 maize races under laboratory conditions (n = 3 with 10 female mites per replicate).

Race	24 h *		48 h		72 h	
	Eggs	Damage	Eggs	Damage	Eggs	Damage
Raton	7.00 ± 5.00 ^b^	13.33 ± 5.77 ^b^	12.67 ± 8.02 ^b^	31.67 ± 7.64 ^a,b^	14.67 ± 9.29 ^b^	56.67 ± 20.82 ^a,b,c^
Olotillo	15.33 ± 10.07 ^a,b^	16.67 ± 5.77 ^a,b^	22.67 ± 17.01 ^a,b^	38.33 ± 16.07 ^a,b^	27.33 ± 20.33 ^a,b^	60.00 ± 26.46 ^a,b,c^
Tuxpeño Norteño	21.00 ± 4.36 ^a,b^	16.67 ± 2.89 ^a,b^	22.33 ± 4.93 ^a,b^	46.67 ± 11.55 ^a,b^	25.67 ± 4.51 ^a,b^	56.67 ± 11.55 ^a,b,c^
Bolita × Raton	17.67 ± 8.74 ^a,b^	26.67 ± 5.77 ^a^	26.67 ± 8.74 ^a,b^	46.67 ± 5.77 ^a,b^	33.33 ± 6.03 ^a,b^	70.00 ± 0.00 ^a^
Elotes Occidentales × Tuxpeño	9.67 ± 5.85 ^b^	26.67 ± 11.55 ^a^	15.33 ± 10.69 ^b^	36.67 ± 20.21 ^a,b^	17.67 ± 10.97 ^b^	45.00 ± 25.98 ^a,b,c^
Tabloncillo × Tuxpeño	13.33 ± 7.37 ^a,b^	18.33 ± 7.64 ^a,b^	17.67 ± 10.69 ^a,b^	25.00 ± 8.66 ^a,b^	20.33 ± 9.29 ^a,b^	25.00 ± 8.66 ^c^
Chalqueño × Tuxpeño	18.67 ± 9.29 ^a,b^	20.00 ± 0.00 ^a,b^	29.00 ± 14.11 ^a,b^	33.33 ± 5.77 ^a,b^	34.00 ± 19.67 ^a,b^	50.00 ± 17.32 ^a,b,c^
Celaya	25.67 ± 17.04 ^a^	26.67 ± 15.28 ^a^	42.67 ± 26.86 ^a^	48.33 ± 29.30 ^a^	49.33 ± 28.54 ^a^	65.00 ± 35.00 ^a,b^
Vandeño	13.33 ± 5.69 ^a,b^	11.67 ± 2.89 ^b^	18.00 ± 7.00 ^a,b^	21.67 ± 7.64 ^b^	20.33 ± 9.29 ^a,b^	30.00 ± 18.03 ^b,c^
Nal Tel × Raton	18.33 ± 12.66 ^a,b^	20.00 ± 8.66 ^a,b^	33.67 ± 30.07 ^a,b^	40.00 ± 25.98 ^a,b^	44.33 ± 40.25 ^a,b^	55.00 ± 32.79 ^a,b,c^
Tuxpeño	10.33 ± 6.81 ^a,b^	16.67 ± 2.89 ^a,b^	13.00 ± 6.56 ^b^	26.67 ± 2.89 ^a,b^	16.33 ± 8.08 ^b^	46.67 ± 5.77 ^a,b,c^

* Means values and ± standard deviation (SD) is presented. Different letters indicate significant differences (*p* < 0.05; MANOVA and LSD’s test).

**Table 5:** Daily (hours = h) changes in growth rate of *Tetranychus merganser* on 11 maize races under laboratory conditions (*n* = 3 with 10 female mites per replicate).

Race	Growth Rate *		
	24 h	48 h	72 h
Raton	0.1482 ± 0.61 ^a^	0.2064 ± 0.36 ^a^	0.1660 ± 0.22 ^a^
Olotillo	0.6804 ± 0.58 ^a^	0.377 ± 0.50 ^a^	0.3074 ± 0.31 ^a^
Tuxpeño Norteño	1.0231 ± 0.23 ^a^	0.5026 ± 0.10 ^a^	0.3617 ± 0.07 ^a^
Bolita × Raton	0.8386 ± 0.46 ^a^	0.5833 ± 0.17 ^a^	0.4524 ± 0.06 ^a^
Elotes Occidentales × Tuxpeño	0.3006 ± 0.59 ^a^	0.2451 ± 0.36 ^a^	0.2013 ± 0.21 ^a^
Tabloncillo × Tuxpeño	0.5736 ± 0.59 ^a^	0.3149 ± 0.43 ^a^	0.2809 ± 0.19 ^a^
Chalqueño × Tuxpeño	0.843 ± 0.47 ^a^	0.5698 ± 0.27 ^a^	0.4139 ± 0.21 ^a^
Celaya	0.9598 ± 0.92 ^a^	0.6984 ± 0.43 ^a^	0.5211 ± 0.25 ^a^
Vandeño	0.5496 ± 0.49 ^a^	0.3869 ± 0.22 ^a^	0.2801 ± 0.17 ^a^
Nal Tel × Raton	0.6898 ± 0.75 ^a^	0.5596 ± 0.43 ^a^	0.4346 ± 0.31 ^a^
Tuxpeño	0.3384 ± 0.53 ^a^	0.2439 ± 0.26 ^a^	0.2074 ± 0.16 ^a^

* Means values and ± standard deviation (SD) is presented. Same letters indicate are not significantly different (*p* < 0.05; MANOVA and LSD’s test).

**Table 6:** Percentage of *Tetranychus merganser* dead on leaf squares of different maize races at different times under laboratory conditions (*n* = 3 with 10 female mites per replicate).

Race	24 h *	48 h	72 h
Raton	40.00 ± 20.00 ^a^	50.00 ± 30.00 ^a^	56.67 ± 32.15 ^a^
Olotillo	33.33 ± 20.82 ^a^	46.67 ± 40.41 ^a^	50.00 ± 40.00 ^a^
Tuxpeño Norteño	26.67 ± 25.17 ^a^	46.67 ± 11.55 ^a^	56.67 ± 15.28 ^a^
Bolita × Raton	30.00 ± 17.32 ^a^	33.33 ± 20.82 ^a^	40.00 ± 17.32 ^a^
Elotes Occidentales × Tuxpeño	46.67 ± 23.09 ^a^	60.00 ± 26.46 ^a^	66.67 ± 25.17 ^a^
Tabloncillo × Tuxpeño	36.67 ± 20.82 ^a^	46.67 ± 37.86 ^a^	46.67 ± 30.22 ^a^
Chalqueño × Tuxpeño	36.67 ± 25.17 ^a^	46.67 ± 35.12 ^a^	46.67 ± 35.12 ^a^
Celaya	30.00 ± 36.06 ^a^	33.33 ± 32.15 ^a^	33.33 ± 32.15 ^a^
Vandeño	46.67 ± 25.17 ^a^	50.00 ± 20.00 ^a^	53.33 ± 15.28 ^a^
Nal Tel × Raton	46.67 ± 30.55 ^a^	46.67 ± 30.55 ^a^	53.33 ± 35.12 ^a^
Tuxpeño	50.00 ± 20.00 ^a^	53.33 ± 15.28 ^a^	63.33 ± 20.82 ^a^

* Means values and ± standard deviation (SD) is presented. Same letters indicate are not significantly different (*p* > 0.05; MANOVA and LSD’s test).

**Table 7:** Eigenvalue and percentage of variances retained by principal components that describe the resistance mechanisms of the 11 races of maize to *Tetranychus merganser* at 24 h under laboratory conditions.

Value	PC1	PC2	PC3	PC4
Eigenvalue	4.26	2.65	1.63	1.37
Percentage of explained variance	35.56	22.06	13.57	11.45
Cumulative variance percentage	35.56	57.63	71.19	82.64

**Table 8:** Eigenvalue and percentage of variances retained by principal components that describe the resistance mechanisms of the 11 races of maize to *Tetranychus merganser* at 48 h under laboratory conditions.

Value	PC1	PC2	PC3	PC4
Eigenvalue	4.51	2.81	1.73	1.28
Percentage of explained variance	37.62	23.42	14.47	10.67
Cumulative variance percentage	37.62	61.04	75.51	86.19

**Table 9:** Eigenvalue and percentage of variances retained by principal components that describe the resistance mechanisms of the eleven races of maize to *Tetranychus merganser* at 72 h under laboratory conditions.

Value	PC1	PC2	PC3	PC4
Eigenvalue	5.23	2.82	1.60	1.37
Percentage of explained variance	40.22	21.68	12.32	10.51
Cumulative variance percentage	40.22	61.91	74.23	84.74

**Table 10:** Race of maize used for the study of resistance to *Tetranychus merganser*.

ID	Race	Location	Latitude	Longitude	MASL
39	Raton	Hidalgo	24.28	−99.42	413
1	Olotillo	Jaumave	23.51	−99.38	783
4	Tuxpeño Norteño	Jaumave	23.39	−99−.41	722
12	Bolita × Raton	Jaumave	23.35	−99.43	846
21	Elotes Occidentales × Tuxpeño	Tula	23.01	−99.62	1291
27	Tabloncillo × Tuxpeño	Tula	22.99	−99.66	1233
51	Chalqueño × Tuxpeño	Palmillas	23.17	−99.56	1549
75	Celaya	Miquihuana	23.46	−99.63	1749
77	Vandeño	Jaumave	23.58	−99.34	656
83	Nal Tel × Raton	Jaumave	23.74	−99.45	841
91	Tuxpeño	Ocampo	22.87	−99.39	407

ID—Identification number in the Germplasm Bank of the Institute of Applied Ecology, Autonomous University of Tamaulipas. MASL—Meters above sea level.

## Data Availability

The data presented in this study are available on request from the corresponding author.

## References

[1] Vielle-Calzada J.P., Padilla J., Bennetzen J.L., Hake S.C. (2009). The Mexican landraces: Description, classification and diversity. Handbook of Maize: Its Biology.

[2] Ureta C., Martinez-Meyer E., Perales H.R., Álvarez-Buylla E.R. (2012). Projecting the effects of climate change on the distribution of maize races and their wild relatives in Mexico. Glob. Change Biol..

[3] González-Martínez J., Rocandio-Rodríguez M., Contreras-Toledo A.R., Joaquín-Cancino S., Vanoye-Eligio V., Chacón-Hernández J.C., Hernández-Bautista A. (2020). Diversidad morfológica y agronómica de maíces nativos del Altiplano de Tamaulipas, México. Rev. Fitotec. Mex..

[4] USDA (2021). World Agricultural Production. Foreign Agricultural Service. Circular Series WAP 7-21. https://apps.fas.usda.gov/psdonline/circulars/production.pdf.

[5] Oerke E.-C. (2006). Crop losses to pests. J. Agric. Sci..

[6] Deutsch C.A., Tewksbury J.J., Tigchelaar M., Battisti D.S., Merrill S.C., Huey R.B., Naylor R.L. (2018). Increase in crop losses to insect pests in a warming climate. Science.

[7] Bui H., Greenhalgh R., Gill G.S., Ji M., Kurlovs A.H., Ronnow C., Lee S., Ramirez R.A., Clark R.M. (2021). Maize inbred line B96 is the source of large-effect loci for resistance to generalist but not specialist spider mites. Front. Plant Sci..

[8] Carena M.J., Glogoza P. (2004). Resistance of maize to the corn leaf aphid: A review. Maydica.

[9] Hassan Y., Abbas N., Li Y., Zhang Y. (2018). Selection for resistance, life history traits and the biochemical mechanism of resistance to thiamethoxam in the maize armyworm, *Mythimna separata* (Lepidoptera: Noctuidae). Phytoparasitica.

[10] Malook S., Xu Y., Qi J., Wang L., Wu J. (2021). *Mythimna separata* herbivory primes maize resistance in systemic leaves. J. Exp. Bot..

[11] Migeon A., Dorkeld F. Spider Mites Web: A Comprehensive Database for the Tetranychidae. http://www1.montpellier.inra.fr/CBGP/spmweb.

[12] Bui H., Greenhalgh R., Ruckert A., Gill G.S., Lee S., Ramirez R.A., Clark R.M. (2018). Generalist and specialist mite herbivores induce similar defense responses in maize and barley but differ in susceptibility to benzoxazinoids. Front. Plant Sci..

[13] Bacon O.G., Lyons T., Baskett R.S. (1962). Effects of spider mite infestations on dent corn in California. J. Econ. Entomol..

[14] Bynum E.D., Michels J., MacDonald J.C., Bible J.B. (2015). Impact of banks grass mite1 damage to yield and quality of maize silage. Southwest. Entomol..

[15] Fathipour Y., Maleknia B., Omkar O. (2016). Mite Predators. Ecofriendly Pest Management for Food Security.

[16] López-Bautista E. (2014). Incidencia de Daño y Estrategias de Control de *Tetranychus merganser* en el Cultivo de Papaya (*Carica papaya* L.). Ph.D. Thesis.

[17] López-Bautista E., Santillán-Galicia M.T., Suárez-Espinosa J., Cruz-Huerta N., Bautista-Martínez N., Alcántara-Jiménez J.A. (2016). Damage caused by mite *Tetranychus merganser* (Trombidiformes: Tetranychidae) on *Carica papaya* (Violales: Caricaceae) plants and effect of two species of predatory mite. Int. J. Acarol..

[18] Smith C.M. (2005). Plant Resistance to Arthropods: Molecular and Conventional Approaches.

[19] Smith C.M., Clement S.L. (2012). Molecular bases of plant resistance to arthropods. Annu. Rev. Entomol..

[20] Kamali K., Dicke F.F., Guthrie W.D. (1989). Resistance-susceptibility of maize genotypes to artificial infestations by twospotted spider mites. Crop Sci..

[21] Franzin M.L., Coffler J.M., Matiello M.A., Ferreira J.O., Mendes S.M. (2020). Multiple infestations induce direct defense of maize to *Tetranychus urticae* (Acari: Tetranychidae). Florida Entomol..

[22] Mansour E., Bar-Zur A., Abo-Moch E. (1993). Resistance of maize inbred lines to the carmine spider mite, *Tetranychus cinnabarinus* (Acari: Tetranychidae): Evaluation of antibiosis of selected lines at different growth stages. Maydica.

[23] Tadmor Y., Lewinsohn E., Abo-Moch F., Bar-Zur A., Mansour E. (1999). Antibiosis of maize inbred lines to the carmine spider mite, *Tetranychus cinnabarinus*. Phytoparasitica.

[24] Chacón-Hernández J.C., Ordaz-Silva S., Mireles-Rodriguez E., Rocandio-Rodríguez M., López-Sánchez I.V., Heinz-Castro R.T.Q., Reyes-Zepeda F., Castro-Nava S. (2020). Resistance of wild chili (*Capsicum annuum* L. var. *glabriusculum*) to *Tetranychus merganser1* Boudreaux. Southwest. Entomol..

[25] Treviño-Barbosa G., Ordaz-Silva S., Gaona-García G., Hernández-Juárez A., Mora-Ravelo S.G., Chacón Hernández J.C. (2022). The resistance of seven host plants to *Tetranychus merganser* Boudreaux (Acari: Tetranychidae). Insects.

[26] Ullah M.S., Moriya D., Badii M.H., Nachman G., Gotoh T.A. (2011). comparative study of development and demographic parameters of *Tetranychus merganser* and *Tetranychus kanzawai* (Acari: Tetranychidae) at different temperatures. Exp. Appl. Acarol..

[27] Reyes-Pérez N., Villanueva-Jiménez J.A., De-la-Cruz-Vargas-Mendoza M., Cabrera-Mireles H., Otero-Colina G. (2013). Parámetros poblacionales de *Tetranychus merganser* Boudreaux (Acari: Tetranychidae) en papayo (*Carica papaya* L.) a diferentes temperaturas. Agrociencia.

[28] Valencia-Domínguez H.M., Otero-Colina G., Santillán-Galicia M.T., Hernández-Castro E. (2011). Acarofauna en papaya var. Maradol (*Carica papaya* L.) en el estado de Yucatán, México. Entomotropica.

[29] Santamaria M.E., Arnaiz A., Gonzalez-Melendi P., Martinez M., Diaz I. (2018). Plant perception and short-term responses to phytophagous insects and mites. Int. J. Mol. Sci..

[30] Shoorooei M., Lotfi M., Nabipour A., Mansouri A.I., Kheradmand K., Zalom F.G., Madadkhah E., Parsafar A. (2013). Antixenosis and antibiosis of some melon (*Cucumis melo*) genotypes to the two-spotted spider mite (*Tetranychus urticae*) and a possible mechanism for resistance. J. Hort. Sci. Biotechnol..

[31] Koul O. (2005). Insect Antifeedants.

[32] Pavela R., Dubey N.K. (2011). Natural products as allelochemicals in pest management. Natural Products in Plant Pest Management.

[33] Marriott J., Florentine S., Raman A. (2013). Effects of *Tetranychus lintearius* (Acari: Tetranychidae) on the structure and water potential in the foliage of the invasive *Ulex europaeus* (Fabaceae) in Australia. Int. J. Acarol..

[34] Bensoussan N., Santamaria M.E., Zhurov V., Diaz I., Grbić M., Grbić V. (2016). Plant-herbivore interaction: Dissection of the cellular pattern of *Tetranychus urticae* feeding on the host plant. Front. Plant Sci..

[35] Najafabadi S.S.M. (2012). Comparative biology and fertility life tables of *Tetranychus urticae* Koch (Acari: Tetranychidae) on different common bean cultivars. Int. J. Acarol..

[36] Paulo P.D., Lima C.G., Dominiquini A.B., Fadini M.A.M., Mendes S.M., Marinho C.G.S. (2018). Maize plants produce direct resistance elicited by *Tetranychus urticae* Koch (Acari: Tetranychidae). Braz. J. Biol..

[37] Najafabadi S.S.M. (2019). Evaluation of cucumber cultivars for resistance to *Tetranychus urticae* Koch. and *Tetranychus turkestani* Ugarov & Nikolski (Acari: Tetranychidae). Songklanakarin J. Sci. Technol..

[38] Najafabadi S.S.M., Bagheri A., Seyahooei M.A. (2019). Cucumber cultivar responses to two tetranychid mites, two-spotted spider mite and strawberry spider mite in greenhouses. Syst. Appl. Acarol..

[39] Puspitarini R.D., Fernando I., Rachmawati R., Hadi M.S., Rizali A. (2021). Host plant variability affects the development and reproduction of *Tetranychus urticae*. Int. J. Acarol..

[40] Ullah F., Lee J.-H., Farhatullh (2006). Evaluation of cucumber (*Cucumis sativus* L.) accessions (cultivars and lines) against the two-spotted spider mite (*Tetranychus urticae* Koch.) and kanzawa spider mite (*T. kanzawai* Kishida, Acari: Tetranychidae). Songklanakarin J. Sci. Technol..

[41] Golizadeh A., Ghavidel S., Razmjou J., Fathi S.A.A., Hassanpour M. (2017). Comparative life table analysis of *Tetranychus urticae* Koch (Acari: Tetranychidae) on ten rose cultivars. Acarologia.

[42] Arnason J.T., Conilh de Beyssac B., Philogene B.J.R., Bergvinson D., Serratos J.A., Mihm J.A., Mihm J.A. (1994). Mechanisms of resistance in maize grain to the maize weevil and the larger grain borer. Insect Resistant Maiz: Recent Advances and Utilization.

[43] Santiago R., Butron A., Reid L.M., Arnason J.T., Sandoya G., Souto X.C., Malvar R.A. (2006). Diferulate content of maize sheaths is associated with resistance to the Mediterranean corn borer *Sesamia nonagrioides* (Lepidoptera: Noctuidae). J. Agric. Food Chem..

[44] McMullen M.D., Frey M., Degenhardt J., Bennetzen J.L., Hake S.C. (2009). Genetics and biochemistry of insect resistance in maize. Handbook of Maize: Its Biology.

[45] Agut B., Gamir J., Jacas J.A., Hurtado M., Flors V. (2014). Different metabolic and genetic responses in citrus may explain relative susceptibility to Tetranychus urticae. Pest. Manag. Sci..

[46] Santamaria M.E., Arnaiz A., Rosa-Diaz I., González-Melendi P., Romero-Hernandez G., Ojeda-Martinez D.A., Garcia A., Contreras E., Martinez M., Diaz I. (2020). Plant defenses against *Tetranychus urticae*: Mind the gaps. Plants.

[47] Zavala-López M., Flint-García S., García-Lara S. (2020). Compositional variation in trans-ferulic, p-coumaric, and diferulic acids levels among kernels of modern and traditional maize (*Zea mays* L.) hybrids. Front. Nutr..

[48] Razmjou J., Vorburger C., Tavakkoli H., Fallahi A. (2009). Comparative population growth parameters of the two-spotted spider mite, *Tetranychus urticae* Koch (Acari: Tetranychidae), on different common bean cultivars. Syst. Appl. Acarol..

[49] González-Martínez J., Rocandio-Rodríguez M., Chacón-Hernández J.C., Vanoye-Eligio V., Moreno-Ramírez Y.R. (2018). Distribución y diversidad de maíces nativos (*Zea mays* L.) en el altiplano de Tamaulipas, México. Agroproductividad.

[50] González-Martínez J., Vanoye-Eligio V., Chacón-Hernández J.C., Rocandio-Rodríguez M. (2019). Diversidad y caracterización de maíces nativos de la Reserva de la Biósfera “El Cielo”, Tamaulipas, México. CienciaUAT.

[51] Ribeiro M.G.P.M., Filho M.M., Guedes I.M.R., Junqueira A.M.R., De-Liz R.S. (2012). Effect of chemical fertilization on two-spotted-spider mite infestation and strawberry yield. Hortic. Bras..

[52] Alizade M., Hosseini M., Modarres-Awal M., Goldani M., Hosseini A. (2016). Effects of nitrogen fertilization on population growth of two-spotted spider mite. Syst. Appl. Acarol..

[53] Singleton V.L., Orthofer R., Lamuela-Raventos R.M. (1999). Analysis of total phenols and other oxidation substrates and antioxidants by means of folin-ciocalteu reagent. Methods Enzymol..

[54] Chang C.-C., Yang M.-H., Wen H.-M., Chern J.-C. (2002). Estimation of total flavonoid content in propolis by two complementary colometric methods. J. Food Drug Anal..

[55] Benzie I.F.F., Strain J.J. (1996). The ferric reducing ability of plasma (FRAP) as measure of “antioxidant power”: The FRAP assay. Anal. Biochem..

[56] Torres-Castillo J.A., Sinagawa-García S.R., Torres-Acosta R.I., García-García L.D., Ramos-Rodríguez A.G., Villanueva-Bocanegra B., Moreno-Ramírez Y.R. (2018). Entomochemicals from *Pterophylla beltrani* Bolivar and Bolivar1: Antioxidants and other metabolites. Southwest. Entomol..

[57] Mrak E.M., Phaff H.J., Mackinney G. (1949). A simple test for carotenoid pigments in yeasts. J. Bacteriol..

[58] Ahmadi A. (1983). Demographic toxicology as a method for studying the dicofol two spotted spider mite (Acari: Tetranychidae) system. J. Econ. Entomol..

[59] Hussey N.W., Parr W.J. (1963). The effect of glasshouse red spider mite (*Tetranychus urticae* Koch) on the yield of cucumbers. J. Hortic. Sci..

[60] Nachman G., Zemek R. (2002). Interactions in a tritrophic acarine predator-prey metapopulation system III: Effects of *Tetranychus urticae* (Acari: Tetranychidae) on host plant condition. Exp. Appl. Acarol..

[61] Birch L.C. (1948). The intrinsic rate of increase of an insect population. J. Anim. Ecol..

[62] Stark J.D., Tanigoshi L., Bounfour M., Antonelli A. (1997). Reproductive potential: Its influence on the susceptibility of a species to pesticides. Ecotoxicol. Environ. Saf..

[63] Everitt B., Hothorn T. (2011). An Introduction to Applied Multivariate Analysis with R.

[64] Kaiser H.F. (1961). A note on Guttman’s lower bound for the number of common factors. Br. J. Stat. Psychol..

[65] Kassambara A. (2017). Practical Guide to Principal Component Methods in R: PCA, (M)CA, FAMD, MFA, HCPC, Factoextra.

[66] R Core Team (2021). R: A Language and Environment for Statistical Computing.

